# Normalization of abnormal plasma amino acid profile-based indexes in patients with gynecological malignant tumors after curative treatment

**DOI:** 10.1186/s12885-018-4875-7

**Published:** 2018-10-12

**Authors:** Yukio Suzuki, Aya Tokinaga-Uchiyama, Taichi Mizushima, Yasuyo Maruyama, Tae Mogami, Nahoko Shikata, Atsuko Ikeda, Hiroshi Yamamoto, Etsuko Miyagi

**Affiliations:** 1 Department of Obstetrics and Gynecology, Yokohama City University Graduate School of Medicine, 3-9 Fukuura, Kanazawa-ku, Yokohama, 236-0004 Japan; 2 Research Institute for Bioscience Products & Fine Chemicals, Ajinomoto Co., Inc, 1-1, Suzuki-cho, Kawasaki-ku, Kawasaki-shi, Kanagawa 210-8681 Japan

**Keywords:** Plasma amino acid profile, Cervical cancer, Endometrial cancer, Ovarian cancer, Curative treatment, Tumor marker

## Abstract

**Background:**

We developed a novel plasma amino acid profile-based index (API) to detect ovarian, uterine, cervical, and endometrial cancers. In this study, we aimed to evaluate whether abnormal API values could be normalized after curative treatment in patients with gynecological malignant tumors.

**Methods:**

Patients with gynecological cancer with abnormal API values were included in this study. Pre-operative absolute API values were compared with those after curative treatment. The normalization rates of API values in patients negative for the expression of three well-known tumor markers (SCC, CA125, and CA19–9) were also evaluated. In addition, related amino acid profiles in healthy controls and patients under pre- and post-treatment conditions were analyzed.

**Results:**

Among 94 patients with abnormal pre-operative API values, the median API value was decreased from 9.52 to 2.17 after treatment (normalization rate: 88.3%). The decreased ranges were similar in patients with adenocarcinoma (6.28; 95% confidence interval [CI]: 5.43–6.95) and squamous carcinoma (7.44; 95% CI: 3.04–8.46). In 93.5% (43/46) of patients negative for tumor markers prior to operation, API values were normalized after the successful treatment. In addition, some pre-operative abnormal amino acid profiles, including Ile, Trp, and His, were reversibly normalized after treatment.

**Conclusion:**

The API is a promising tumor marker in gynecological malignancies for the diagnosis of remission, particularly in patients negative for general tumor markers. Further studies are needed to explore the mechanisms related to the normalization of abnormal amino acid profiles.

**Electronic supplementary material:**

The online version of this article (10.1186/s12885-018-4875-7) contains supplementary material, which is available to authorized users.

## Background

According to the 2016 cancer registry and statistics, the screening rate of cervical cancer in Japan is still as low as 42.3%, and the incidences of endometrial and ovarian cancer are in increasing trends [1]. For these gynecological cancers, various serum markers have been developed and used; however, the sensitivity and specificity of these markers are not high, and these markers are not specific for uterine and ovarian cancers. Therefore, the development of specific serum markers for screening without internal examination is urgently needed, particularly for patients with ovarian cancer, who typically do not present any symptoms until an advanced stage.

CA125 was first reported as a tumor marker to detect ovarian tumors in the 1980s [2]. The American College of Obstetricians and Gynecologists recommends CA125 as an effective serum tumor marker, although CA125 lacks the sensitivity and specificity needed to function alone as a screening test [3]. Moreover, CA125 levels are increased in cases of benign ovarian tumors or during menses, which can lead to false-positive results. As recently reported, in patients with gynecological cancers, the accuracy of CA125 analysis has increased by combining this marker with several other serum tumor markers (e.g., HE4) and ultrasound sonography [4]; however, it is still difficult to identify malignancy by serologic diagnosis alone.

The plasma amino acid concentrations of healthy individuals are usually controlled in a certain range by the homeostatic function of the body; however, the levels of various amino acids are altered in patients with different types of cancer, including cervical [5], endometrial [6], ovarian [7], lung [8], breast [9], esophageal [10, 11], colon [9], gastric [9], pancreatic [12], and prostate [9] cancers. Plasma amino acid profile-based indexes (APIs) have already been developed as early screening tests for nine of these cancers (gastric, colon, pancreatic, lung, prostate, breast, cervical, endometrial, and ovarian cancers). These indexes are based on a multivariate analysis of the plasma free amino acid concentration, comparing patients with cancer to healthy controls, and have been introduced as part of comprehensive medical examinations in Japan via AminoIndex Cancer Screening (AICS; Ajinomoto Co., Inc., Tokyo, Japan).

For ovarian, cervical, and endometrial cancers, alterations in plasma amino acid profiles are similar; therefore, a single API was developed to include all of these cancers (AICS (uterine/ovarian)) [6, 7]. Using this API, we have evaluated the cutoff value needed to distinguish benign and malignant tumors for the diagnosis of ovarian cancer in comparison with CA125. When a cutoff value of 6.0 or more was used, the positive rate for epithelial ovarian cancer was 0.73, which was equivalent to that of CA125, and the false-positive rate for benign endometriotic cyst was 0.35, which was significantly lower than that of CA125 [13].

For patients with gynecological cancer whose API value exceeds the cutoff value, it is not yet clear how API values change after treatment. Therefore, in this study, we examined changes in pre-operative and post-operative API values in patients with cervical cancer, uterine endometrial cancer, and ovarian cancer who underwent surgery and achieved complete remission after primary therapy. The aim of this study was to evaluate whether abnormal API values could be normalized after curative treatment in patients with gynecological malignant tumors with the cutoff value established in our previous study [13].

## Methods

### Study population

We included patients who underwent surgery for gynecologic cancers, including epithelial ovarian cancers, cervical cancers, and uterine endometrial cancers, at the Department of Obstetrics and Gynecology of Yokohama City University Hospital between November 2006 and December 2012 and whose pre-operative API was 6.0 or more. Patients continued to visit our hospital as outpatients, and we performed follow-up blood tests to assess changes in API values from February 2013 to December 2014. In this study, an API of 6.0 or more was defined as an abnormal API according to a previous study [13]. We selected 94 patients who showed no recurrence after curative treatment. Pre-operative absolute API values were compared with those after the curative treatments. The normalization rates of API in patients who were negative for three well-known tumor markers (SCC, CA125, and CA19–9) were also evaluated. The institutional ethics committee approved this study protocol as an industry-academia collaborative project. We collected informed consent forms from all patients.

We used control group data obtained in a previous study [7] for receiver operating characteristic (ROC) curve analysis of each amino acid.

### Sample collection and plasma amino acid analysis

After an overnight fast, blood samples (5 mL) were collected in tubes containing ethylenediaminetetraacetic acid disodium salt (Terumo, Tokyo, Japan) and immediately cooled with ice. Plasma was separated by centrifugation at 2000×g and 4 °C for 15 min and stored at − 80 °C until analysis. After thawing, the plasma samples were deproteinized using acetonitrile at a final concentration of 80% before measurement of amino acid concentrations by high-performance liquid chromatography electrospray ionization/mass spectrometry with precolumn derivatization.

### Plasma API

The API was originally developed as AminoIndex Cancer Screening (AICS; Ajinomoto Co., Inc., Tokyo, Japan), and AICS (uterine/ovarian) is a screening marker for cervical, endometrial, and ovarian cancers based on a multivariate analysis of the plasma free amino acid concentration, comparing patients with cancer to healthy controls. The formula for API includes the following amino acids: Trp, His, Cit, Val, Ile, and Gly. API values were measured by applying concentrations of amino acids as previously described [6] at the Institute for Innovation, Ajinomoto Co., Inc.

### Statistical analysis

To determine the significance of differences in API values before and after surgery, Wilcoxon signed rank tests were used. Chi-squared tests were used to compare API normalization rates in the three gynecological cancers. ROC curve analysis of each amino acid was used to express their capabilities to discriminate between patients with cervical, endometrial, or ovarian cancer and healthy controls, and the area under the curve (AUC) was calculated. All statistical analyses were performed using R version 3.3.0 (R Foundation for Statistical Computing, Vienna, Austria) and GraphPad Prism version 6 (GraphPad Software, Inc., San Diego, CA, USA).

## Results

Of the 94 cases in this study, 22 (23.4%) cases were cervical cancers, 53 (56.4%) cases were endometrial cancers, and 19 (20.2%) cases were ovarian cancers. An average of 54.8 ± 23.1 months (range: 10–95 months) had passed since the operation at the time of postoperative blood tests.

The characteristics of the patients are shown in Table 1. The median age of this cohort was 56 years (range, 24–76 years). Fifty-eight of the 94 patients (61.7%) had stage I disease, 17 (18.1%) had stage II disease, 16 (17.0%) had stage III disease, and three (3.2%) had stage IV disease. Histology types were as follows: 79 cases (84.0%) were adenocarcinoma, 11 cases (11.7%) were squamous cell carcinoma (SCC), and four cases (4.3%) were others. Most cases of SCC were cases of cervical cancers (10 cases). The rate of adjuvant chemotherapy was 56.4% in total.

**Table 1:** Descriptive status of patients

	No. of patients (%)							
	Total		Cervical cancer		Endometrial cancer		Ovarian cancer	
Number of patients	94		22		53		19	
Age at pre-operative API measurement								
Median	56		48		58		56	
Range	24–76		28–72		35–76		37–74	
Stage								
I	58	(61.7)	9	(40.9)	35	(66.0)	14	(73.7)
II	17	(18.1)	12	(54.5)	4	(7.5)	1	(5.3)
III	16	(17.0)	0	(0.0)	13	(24.5)	3	(15.8)
IV	3	(3.2)	1	(4.5)	1	(1.9)	1	(5.3)
Histopathological type								
Adenocarcinoma	79	(84.0)	12	(54.5)	50	(94.3)	17	(89.5)
Endometrioid	45		1		40		4	
Serous	4		0		1		3	
Clear cell	9		0		3		6	
Mucinous	11		7		1		3	
Adenosquamous	4		0		4		0	
NOS	6		4		1		1	
SCC	11	(11.7)	10	(45.5)	1	(1.9)	0	(0.0)
Others	4	(4.3)	0	(0.0)	2	(3.8)	2	(10.5)
Treatment								
Complete surgery	41	(43.6)	7	(31.8)	32	(60.4)	2	(10.5)
Complete surgery + Adj	53	(56.4)	15	(68.2)	21	(39.6)	17	(89.5)

*SCC* squamous cell carcinoma, *NOS* not otherwise specified, *Adj* adjuvant therapy

Table 2 shows changes in API values in each cancer before (pre) and after (post) complete surgery. API values decreased below the cutoff level in 88.3% of all cases; however, there were 11 patients (11.7%) whose API values did not decrease below the cutoff level. The rates of API normalization did not differ significantly among the three gynecological cancers.

**Table 2:** Changes in API before (pre) and after (post) complete surgery

	No. of patients (pre API ≥ 6)	API normalized cases (post API < 6)	Normalization rate
Total	94	83	88.3%
Cervical cancer	22	20	90.9%
Endometrial cancer	53	46	86.8%
Ovarian cancer	19	17	89.5%

**Fig. 1:**
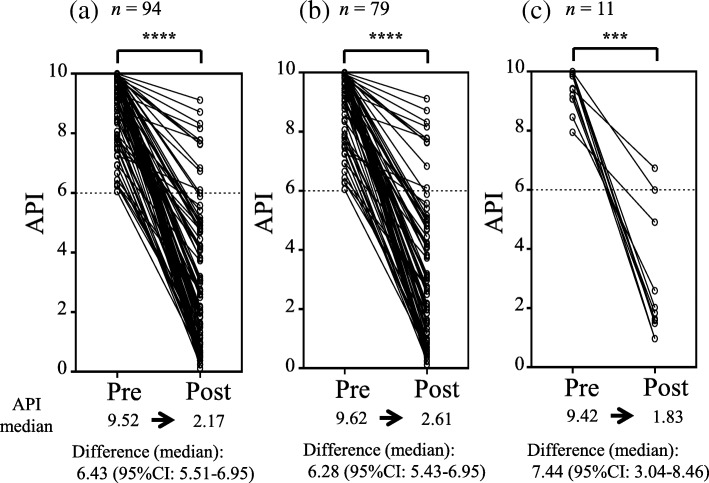
API before (pre) and after (post) complete surgery. (**a**) All patients (*n* = 94), (**b**) patients with adenocarcinoma (*n* = 79), (**c**) patients with squamous cell carcinoma (*n* = 11). Wilcoxon signed rank test, *** *p* < 0.001, **** *p* < 0.0001

Figure 1 shows individual changes in API values in all patients (Fig. 1-a), patients with adenocarcinoma (Fig. 1-b), and patients with SCC (Fig. 1-c) before (pre) and after (post) surgery. API values were normalized in 87.3% of patients with adenocarcinoma, and 90.9% of patients with SCC. The median API decreased significantly from 9.52 before surgery to 2.17 after surgery (*p* < 0.0001), and the median difference was 6.43 (95% confidence interval [CI]: 5.51–6.95) for all patients. A reversible decrease in API value after surgery was observed independent of histology, and the median differences in API before and after surgery were 6.28 (95% CI: 5.43–6.95) for adenocarcinoma and 7.44 (95% CI: 3.04–8.46) for SCC.

Changes in API before (pre) and after (post) complete surgery for patients with negative tumor markers before surgery are shown in Table 3. For patients with undetectable pre-operative levels of SCC, CA125, carcinoembryonic antigen (CEA), or CA19–9, the decrease rates of API values were 85.0%, 90.0%, 89.9%, and 90.5%, respectively (Table 3-a). Even in cases in which the three adenocarcinoma markers CEA, CA19–9, and CA125 were all negative before surgery, API values decreased in 93.5% of patients. For patients with adenocarcinoma who had negative pre-operative CA125, which is the most commonly used tumor marker for adenocarcinoma, 47 out of 52 patients (90.4%) showed decreased API values (Table 3-b).

**Table 3:** Changes in API before and after surgery for patients with negative TM levels before surgery

(a)				
Negative TM presurgery	Histology	No. of patients (pre API ≥ 6)	No. of API normalized cases (post API < 6)	Normalization rate
SCC	All	40	34	85.0%
CA125	All	60	54	90.0%
CEA	All	79	71	89.9%
CA19–9	All	63	57	90.5%
CEA, CA19–9, CA125	All	46	43	93.5%
(b)				
Negative TM presurgery	Histology	No. of patients (pre API ≥ 6)	No. of API normalized cases (post API < 6)	Normalization rate
SCC	Squamous cell Carcinoma	1	1	100%
CA125	Adenocarcinoma	52	47	90.4%

*TM* tumor marker

**Fig. 2:**
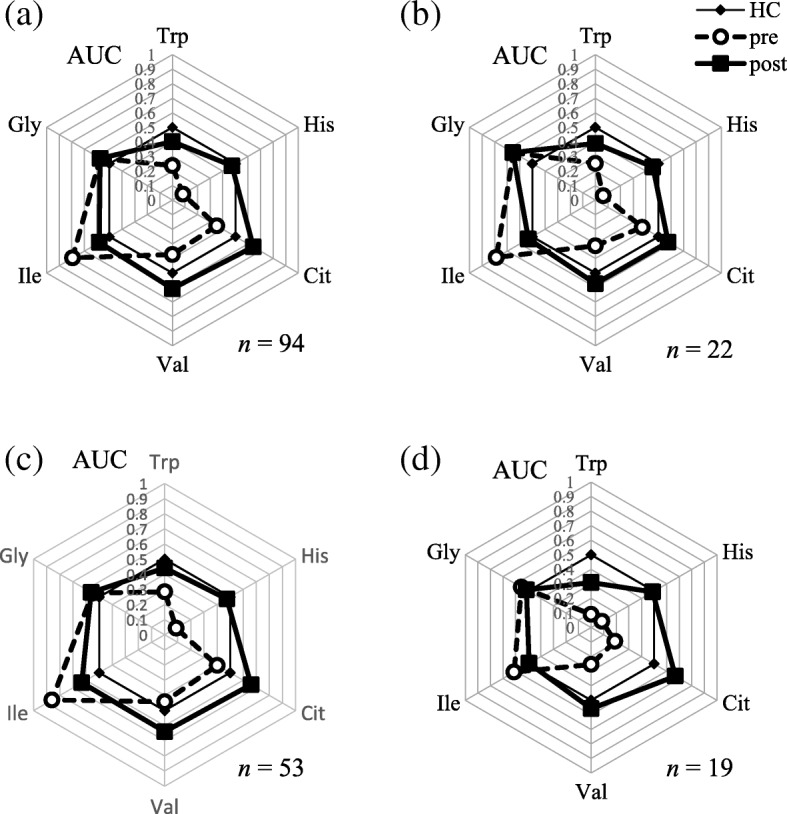
Amino acid profiles of patients with cancer. The results of receiver-operator characteristic curve (ROC) analysis of plasma amino acids before and after complete surgery. Area under the ROC (AUC) for amino acids used to calculate API values are shown for all patients (n = 94) (**a**), patients with cervical cancer (*n* = 22) (**b**), patients with endometrial cancer (*n* = 53) (**c**), and patients with ovarian cancer (*n* = 19) (**d**). HC, healthy control; pre, pre-surgery; and post, post-surgery. Axes show the AUC of each amino acid

We performed ROC curve analysis to determine whether each amino acid concentration in patients was distinguishable from that in healthy controls, and examined the change in each amino acid before and after complete surgery (Additional file 1: Figure S1). The AUC of the ROC curve for each amino acid is shown in Fig. 2. From these data, we visually confirmed that the balance of amino acid profiles approached the standard hexagon shape of the healthy control after surgery, regardless of cancer type. Abnormally low concentrations of His in patients with cancer increased and returned to the level of the healthy control after surgery, and the concentrations of Trp and Ile also began to recover to normal levels. The concentrations of Gly, Cit, and Val did not recover.

## Discussion

In this study, we observed normalization of the API values by curative surgery in 88.3% of patients who had abnormally high API values before treatment. We also observed that a high proportion of patients with negative pre-operative levels of common tumor markers showed decreased API values after surgery. These results indicated that the API could be another useful tumor marker in gynecological malignant tumors.

Alterations of plasma free amino acid profiles in patients with cancer have been reported in many previous studies [5–14]. In particular, plasma free amino acid profiles of ovarian cancers and endometrial cancers have been reported by our study group [6, 7], and we proposed a cutoff to discriminate ovarian tumors from benign tumors [13]. However, most studies have only indicated the usefulness of plasma free amino acid profiles for early cancer detection or discrimination from benign tumors by comparing with healthy or benign control groups. Indeed, few reports have described how plasma free amino acid profiles change after treatment or relapse. In a study of 77 cases of childhood cancer, the usefulness of plasma free amino acid profiles as a biomarker was examined, and this marker was suggested to predict recurrence [14]. In another study describing peri-operative dynamics of plasma free amino acid profiles in patients with cancer, alterations in plasma free amino acid profiles after surgery were found to predict diagnosis in patients with cancer [15]. To the best of our knowledge, this is the first report showing changes in plasma free amino acid profiles during the follow-up period after treatment, and therefore, we believe that our current findings are valuable.

Another important point in this study is that API normalization by surgical treatment was also observed in patients with negative pre-operative tumor marker levels. In clinical practice, the most commonly used tumor marker in ovarian and endometrial cancer is CA125. Previous studies have reported the sensitivity of CA125 to be about 85.1% [16]; therefore, in about 15% of patients, CA125 is not useful as a monitoring marker after surgery. For patients in whom existing tumor markers could not be used, API could be an alternative follow-up biomarker. Recent advances in diagnostic imaging technologies have enabled earlier detection of recurrent tumors; however, blood tests for detecting signs of recurrence with minimal invasion and cost are still needed.

As previously reported, API was first developed as a blood test for gastric, lung, colon, prostate, and breast cancers based on the differences in plasma free amino acids in patients with cancer and healthy controls [9]. We then developed AICS (uterine/ovarian) based on the changes in six amino acid profiles [6]. These previous studies reported the statistically significant changes in specific amino acid concentrations in patients with cancer. However, this study is the first report to show time-dependent changes in individual patients, without a comparison of the two groups. The increased level of Ile before treatment was decreased, and decreased levels of Trp, His, Cit, and Val were increased towards the level of the healthy control after treatment. This is a very important result supporting the hypothesis that the tumor itself causes alterations in plasma amino acid profiles.

In this study, we applied a cutoff value of 6.0 for API values and found that the decrease rate of the API was similar in all cancer types and tumor histology. In a previous study, the cutoff value was evaluated only for ovarian cancer diagnosis [13]. However, our result suggested that a cutoff value of 6.0 was reasonable for using API as a follow-up tumor marker in cervical and endometrial cancers as well as ovarian cancer. We will validate this cutoff value by accumulating more data for patients with recurrent tumors.

There were some limitations to this study. First, we showed that API values decreased following surgical treatment; however, we still do not have data demonstrating when abnormal API values are normalized or how API values change when patients have recurrent tumors. Therefore, we need to carefully consider the usefulness of API as a recurrent monitoring marker. We are continuing to follow-up with patients and collecting data to determine whether API values increase when recurrent tumors are detected. Another limitation is that this study was carried out at a single institution with a relatively small number of patients. For analysis of recurrence markers, it will be necessary to evaluate more patients. The mechanisms underlying alterations in API values due to cancer are still not clear. Indoleamine 2,3-dioxygenase, which catalyzes Trp degradation, has been reported to be involved in the immune escape of tumor cells by depleting Trp [17, 18]. However, the detailed mechanisms explaining the alterations in each amino acid used in the API formula still need to be investigated.

## Conclusions

In this study, we demonstrated that abnormal API values could be normalized after successful surgical treatment. In addition, we observed patients with negative pre-operative levels of common tumor markers also showed decreased API values after curative surgery. From these results, we conclude that the API could be another useful tumor marker in gynecological malignant tumors. The possible application of the API as a tumor marker to monitor time-dependent changes should be explored in future studies.

## Additional file

Additional file 1: **Figure S1.** ROC curve and AUC for each amino acid concentration before (pre) and after (post) complete surgery. ROC curve analysis was performed for each plasma amino acid concentration in healthy controls and patients before and after surgery. The same healthy control group was used for analysis of all patients (a), cervical cancer patients (b), endometrial cancer patients (c), and ovarian cancer patients (d). The AUC was calculated and is indicated in each graph. (PDF 765 kb)

## Abbreviations

AICS: AminoIndex Cancer Screening
API: Plasma amino acid profile-based index
ROC: Receiver operating characteristic
SCC: squamous cell carcinoma
TM: tumor marker
