# Supplementary material for: Normalization of abnormal plasma amino acid profile-based indexes in patients with gynecological malignant tumors after curative treatment
Source: BMC Cancer. 2018 Oct 12;18:973. doi: 10.1186/s12885-018-4875-7 (PMC6186072; doi:10.1186/s12885-018-4875-7)

Figure S1
ROC curve and AUC for each amino acid concentration before (pre) and after (post) complete surgery. ROC curve analysis was performed for each plasma amino acid concentration in healthy controls and patients before and after surgery. The same healthy control group was used for analysis of all patients (a), cervical cancer patients (b), endometrial cancer patients (c), and ovarian cancer patients (d). The AUC was calculated and is indicated in each graph.

(a-1) All patients, pre Trp

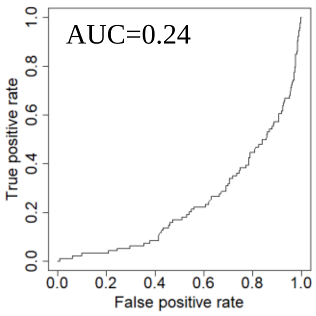

(a-2) All patients, pre His

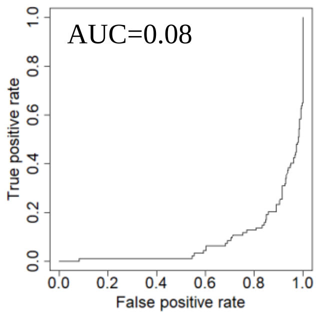

(a-3) All patients, pre Cit

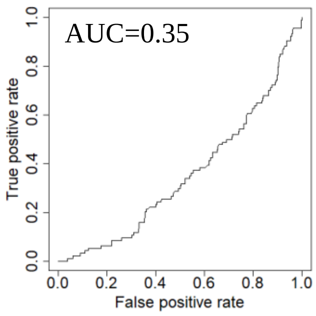

(a-4) All patients, pre Val

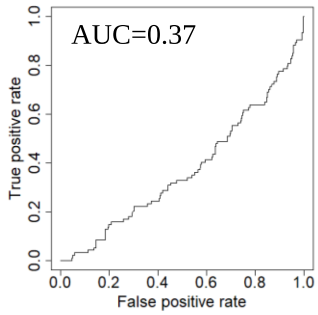

(a-5) All patients, pre Ile

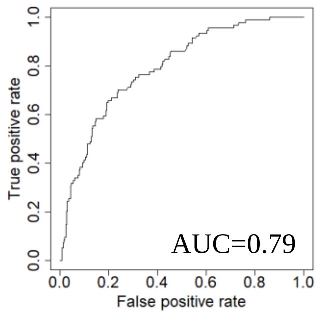

(a-6) All patients, pre Gly

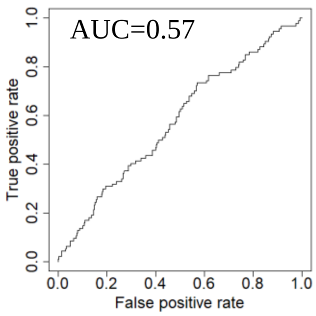

(a-7) All patients, post Trp

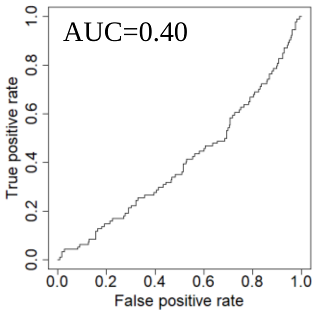

(a-8) All patients, post His

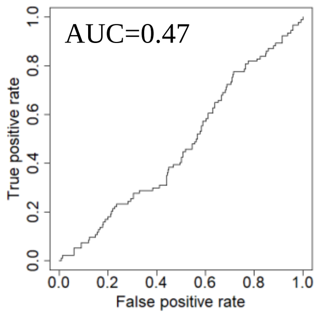

(a-9) All patients, post Cit

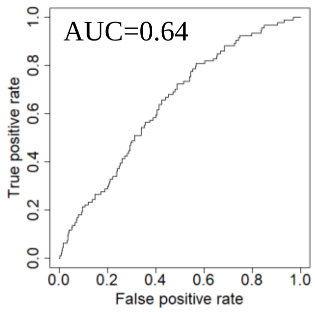

(a-10) All patients, post Val

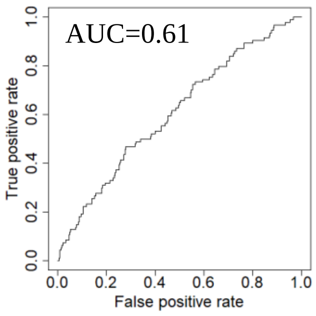

(a-11) All patients, post Ile

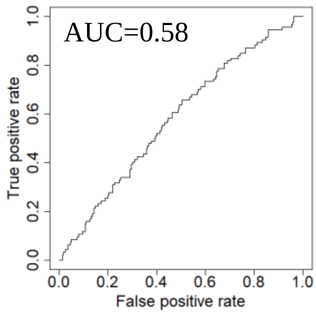

(a-12) All patients, post Gly

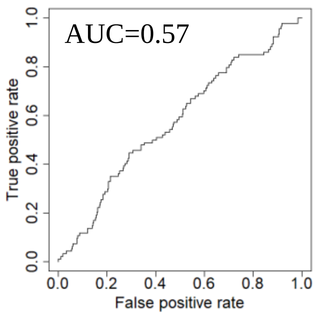

(b-1) Cervical cancer, pre Trp

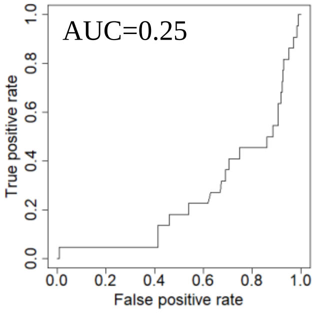

(b-2) Cervical cancer, pre His

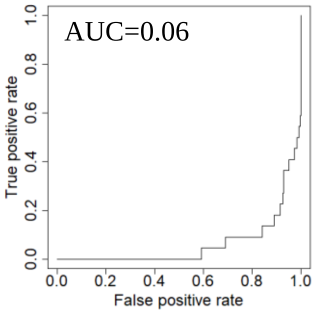

(b-3) Cervical cancer, pre Cit

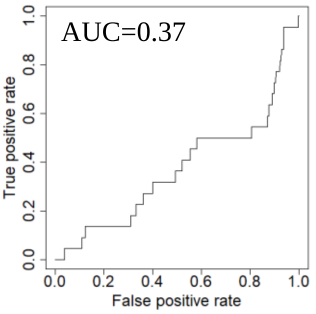

(b-4) Cervical cancer, pre Val

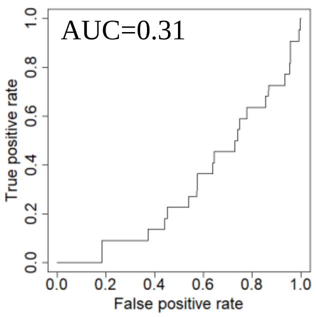

(b-5) Cervical cancer, pre Ile

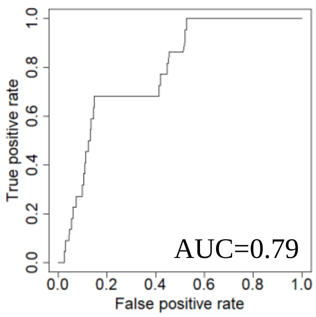

(b-6) Cervical cancer, pre Gly

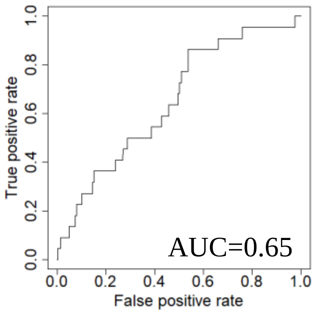

(b-7) Cervical cancer, post Trp

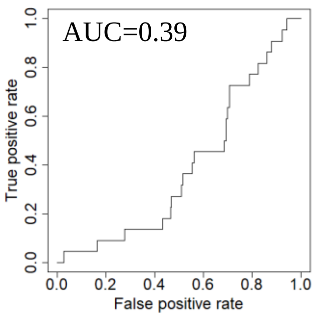

(b-8) Cervical cancer, post His

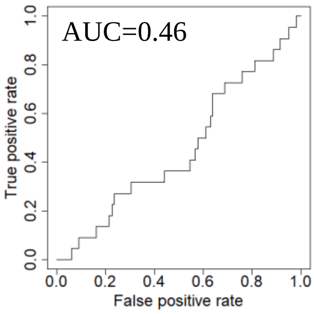

(b-9) Cervical cancer, post Cit

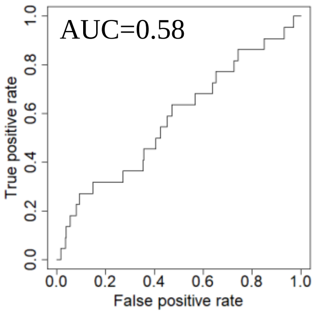

(b-10) Cervical cancer, post Val

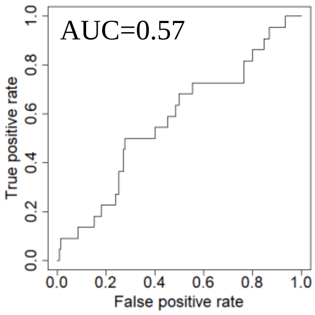

(b-11) Cervical cancer, post Ile

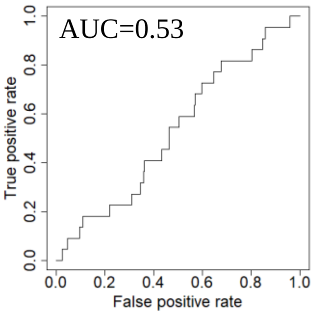

(b-12) Cervical cancer, post Gly

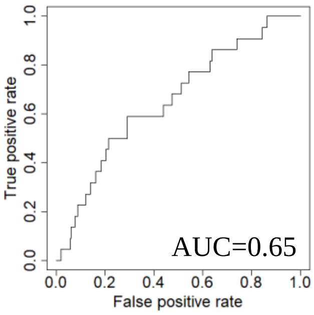

(c-1) Endometrial cancer, pre Trp

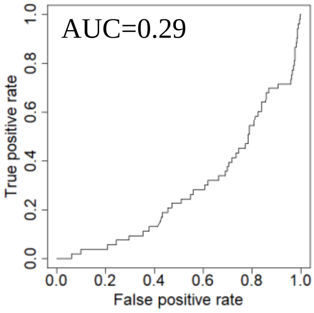

(c-2) Endometrial cancer, pre His

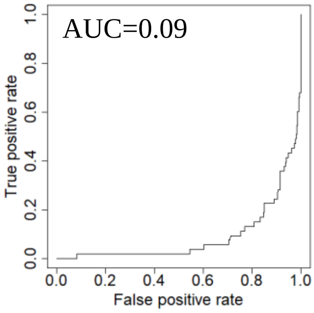

(c-3) Endometrial cancer, pre Cit

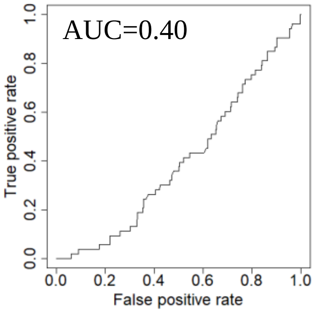

(c-4) Endometrial cancer, pre Val

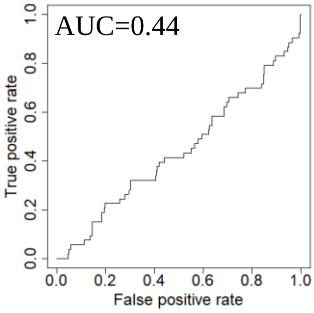

(c-5) Endometrial cancer, pre Ile

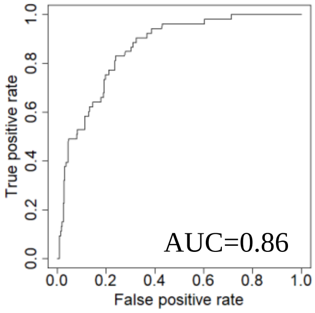

(c-6) Endometrial cancer, pre Gly

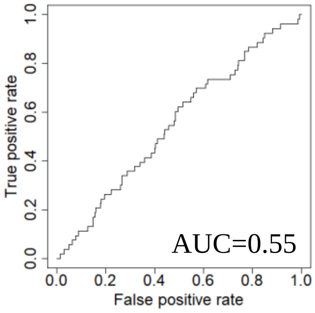

(c-7) Endometrial cancer, post Trp

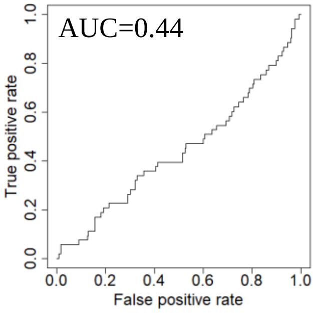

(c-8) Endometrial cancer, post His

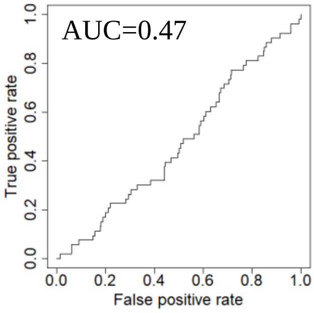

(c-9) Endometrial cancer, post Cit

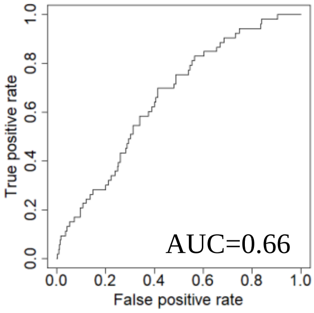

(c-10) Endometrial cancer, post Val

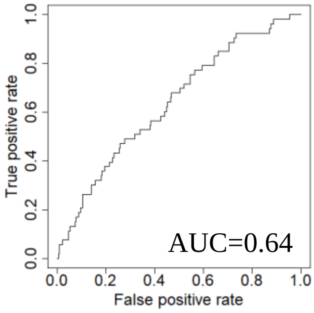

(c-11) Endometrial cancer, post Ile

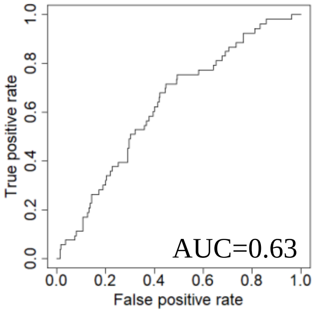

(c-12) Endometrial cancer, post Gly

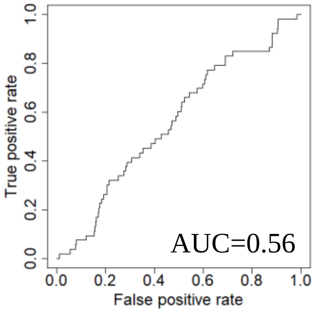

(d-1) Ovarian cancer, pre Trp

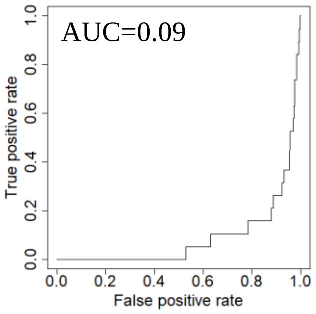

(d-2) Ovarian cancer, pre His

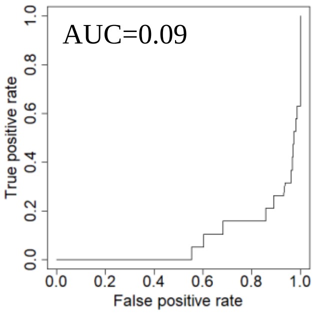

(d-3) Ovarian cancer, pre Cit

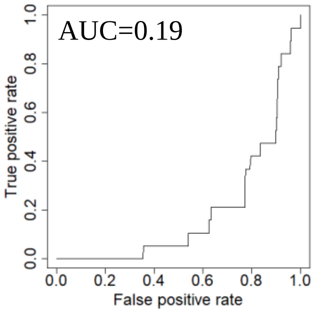

(d-4) Ovarian cancer, pre Val

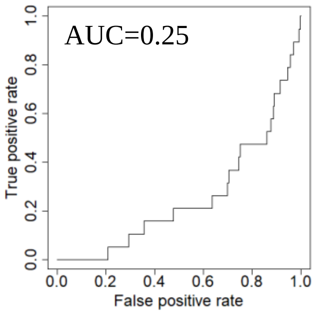

(d-5) Ovarian cancer, pre Ile

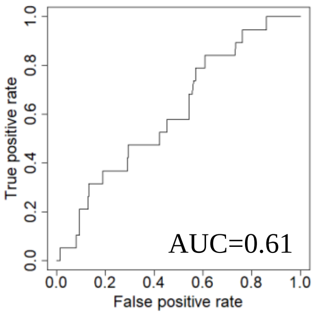

(d-6) Ovarian cancer, pre Gly

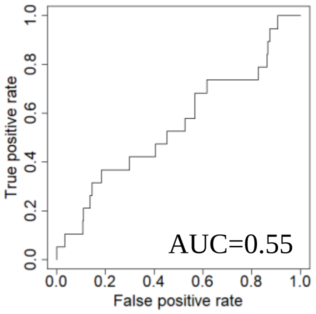

(d-7) Ovarian cancer, post Trp

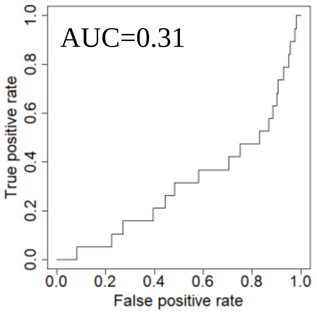

(d-8) Ovarian cancer, post His

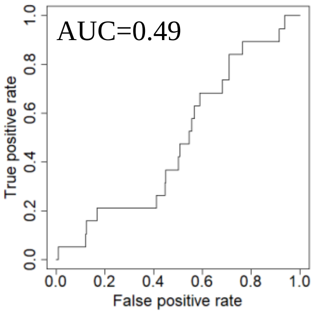

(d-9) Ovarian cancer, post Cit

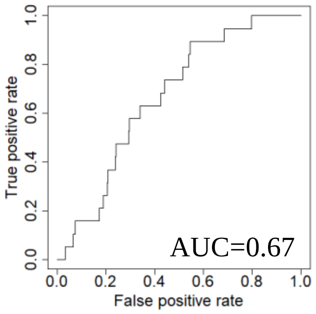

(d-10) Ovarian cancer, post Val

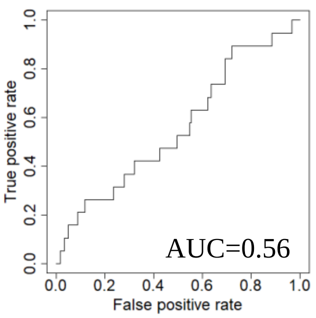

(d-11) Ovarian cancer, post Ile

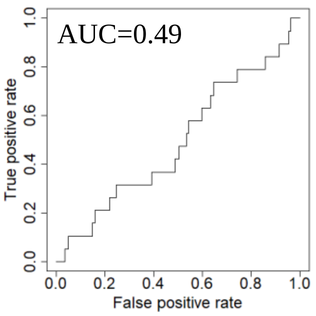

(d-12) Ovarian cancer, post Gly

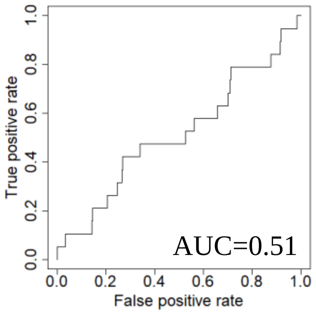

Supplement: Supplementary file 1 — Figure S1. ROC curve and AUC for each amino acid concentration before (pre) and after (post) complete surgery. ROC curve analysis was performed for each plasma amino acid concentration in healthy controls and patients before and after surgery. The same healthy control group was used for analysis of all patients (a), cervical cancer patients (b), endometrial cancer patients (c), and ovarian cancer patients (d). The AUC was calculated and is indicated in each graph. (PDF 765 kb) [file 12885_2018_4875_MOESM1_ESM.pdf]
